# From reference epigenomes to response epigenomes: a public-health case built on the developing brain

**DOI:** 10.3389/fpubh.2026.1916784

**Published:** 2026-09-18

**Authors:** Thomas Hartung

**Affiliations:** 1 Center for Alternatives to Animal Testing (CAAT), Department of Environmental Health and Engineering, Johns Hopkins Bloomberg School of Public Health, Baltimore, MD, United States; 2 CAAT-Europe, University of Konstanz, Konstanz, Germany

**Keywords:** developmental neurotoxicity, DNA methylation, environmental health, epigenetic biomarkers, exposome, gene–environment interaction, precision prevention, response epigenome

## Abstract

Reference epigenome projects, such as the NIH roadmap, ENCODE, and the international human epigenome consortium, have started mapping the baseline regulatory states of human cell types with extraordinary detail. However, these projects were not designed to address the urgent questions posed by exposome science and child-health policy: how these states *change* after environmental exposure, in whom, during which developmental window, and whether they can recover. I argue that the missing object is a human response epigenome: the cell-type-specific change in epigenomic state over time following a defined exposure or mixture, conditioned on internal dose, genotype, developmental stage, sex, ancestry, prior exposure, and time since exposure. Developmental neurotoxicity is the sentinel case for building it: the developing brain is the most vulnerable target organ, neurodevelopmental disorders are rising, several of the highest-confidence genetic risk factors are themselves components of the epigenetic machinery, and the prevention of childhood chronic disease is now an explicit policy priority. I distinguish the response epigenome from the reference and disease epigenomes, define its mapping unit, specify minimum standards and a tiered, sentinel-exposure pilot for its construction, and set the causal and ethical bar it must clear. The claim is deliberately bound: the epigenome is a principal, not the sole, durable interface between genome and environment. Mapping its *responses*, beginning with the developing brain, is the tractable next step toward prevention.

## 1 The question reference epigenomes were not built to answer

Two decades of investment have given biomedicine a remarkable resource: reference maps of the human epigenome. The NIH Roadmap Epigenomics Program produced 111 reference epigenomes and showed that regulatory landscapes are profoundly tissue-specific and that disease-associated genetic variants are enriched in cell-type-relevant marks (1). ENCODE cataloged candidate regulatory elements across human tissues (2). The International Human Epigenome Consortium (IHEC)^1^ coordinated these and other national programs around a common goal, i.e., high-resolution reference epigenomes of major primary cell types, together with assay, quality, and metadata standards, and now serves thousands of datasets (3). Achievement is real and foundational.

But these projects answered one question: *what regulatory states exist in human cell types?* They were never designed to answer another that is now more urgent for public health: *how do those states change after environmental exposure, in whom, during which window of life, and do they recover?* The framing of the epigenome as the layer through which genome and environment are integrated is itself longstanding (4, 5); the exposome concept gave the environmental side of that integration a name and a research program (6–8). What is still missing is not another statement of the bridge or another static map, but a standardized account of the epigenomic *response* (Figure 1). That gap, and a tractable way to begin closing it, is the subject of this perspective.

**Figure 1:**
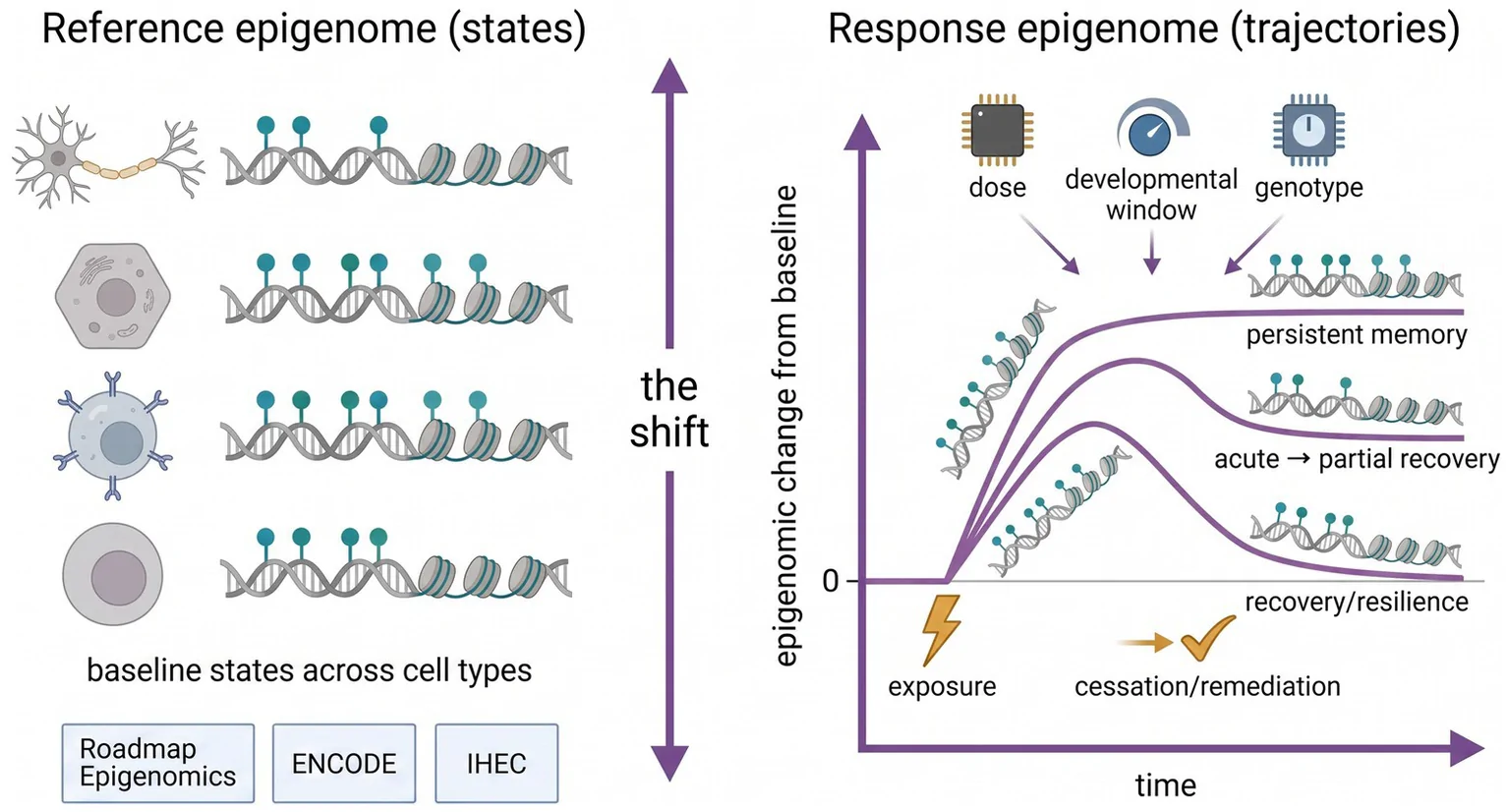
From reference epigenomes to response epigenomes. (Left) Reference epigenome projects (Roadmap, ENCODE, IHEC) mapped the baseline regulatory states of human cell types as a static atlas, answering the question “*what states exist*.” Each cell type carries a fixed pattern of DNA methylation and histone marks. (Right) A response epigenome instead captures how a cell-type-specific epigenomic state changes over time after a defined exposure: a baseline gives way to an acute response, which may resolve back toward baseline (recovery/resilience) or settle into a persistent memory state, with the trajectory conditioned on dose, developmental window, and genotype. The shift in question is not from one map to a better map but from states to trajectories, which is the object public-health prevention requires.

A scope note belongs at the outset, because overclaiming has dogged this field. The epigenome is not the only route from environment to phenotype. Exposures also act through DNA and protein adducts and mutations, receptor binding, metabolic and mitochondrial disruption, oxidative stress, inflammation, and immune modulation, several of which alter cell behavior without first establishing a stable epigenetic state. In cancer, the International Agency for Research on Cancer (IARC^2^) organizes mechanistic evidence for approximately 10 key characteristics of carcinogens, of which “*induces epigenetic alterations*” is one, among genotoxicity, receptor-mediated effects, immunosuppression, and others (9). The epigenome’s distinction lies not in exclusivity but in *durability and cell-state specificity*: it is the principal layer through which a transient exposure can become a persistent, mappable regulatory feature. It is precisely that persistence that makes a *response* atlas worth building and worth building well. In this context, the partnership between the Human Cell Atlas^3^ and the Global Exposome Forum^4^ under the umbrella of UNESCO^5^^,^^6^ is a remarkable opportunity.

## 2 Why now, and why is the developing brain

The case for urgency, and for a developmental entry point, is strongest in child health. Neurodevelopmental disorders have risen in recorded prevalence; recent United States surveillance reports^7^ indicate approximately 1 in 31 8-year-olds with autism spectrum disorder (ASD) (10), and attention-deficit/hyperactivity disorder has climbed in parallel (11). The burden attributable to developmental chemical exposure is quantifiable and large: an analysis of United States exposure to polybrominated diphenyl ethers, organophosphates, methylmercury, and lead estimated, across recent years, the loss of on the order of 162 million IQ points and some 738,000 cases of intellectual disability, with the associated costs of intellectual disability falling from approximately $159 billion to approximately $50 billion annually as exposures to legacy chemicals declined, a decline that itself illustrates the prevention dividend, and a figure shadowed by the rising contribution of replacement chemicals (11). Endocrine disruption, particularly of the thyroid-hormone system, is among the recognized contributors to developmental neurotoxicity (11). Against this, very few of the tens of thousands of chemicals in commerce have been tested for developmental neurotoxicity at all, because the animal guideline study is prohibitively costly: on the order of $1.4 million and more than a thousand animals per substance, over approximately 2 years (11). The prevention of childhood chronic disease, with explicit attention to environmental chemicals and endocrine-disrupting compounds and their links to developmental disorders, has meanwhile moved to the center of health policy: the United States *Make Our Children Healthy Again* assessment names chemical exposure among the principal drivers of childhood chronic disease (12), and the National Institute of Environmental Health Sciences has framed “*epigenetics and the exposome*” as a new era of disease prevention^8^.

Whatever one’s view of the politics (Section 7), the scientific opportunity is clear: prevention requires knowing which exposures perturb development, through which mechanisms, in which windows.

The developing brain is the natural sentinel for three reasons. First, it is the most sensitive target organ in toxicology, built through tightly timed key neurodevelopmental processes (proliferation, migration, differentiation, neurite outgrowth, synaptogenesis, myelination) in narrow, largely non-recoverable windows (11). Second, those windows coincide with the period in which the brain’s own epigenome is being written, as progenitors commit to neuronal and glial fates through chromatin remodeling; an exposure that perturbs the writing machinery during this window has outsized and lasting consequences, a principle established for periconceptional nutritional deprivation (13) and for prenatal tobacco exposure, which leaves reproducible methylation signatures in newborn blood (14). Third, and most striking, the genetics of ASD are substantial but incomplete (inherited variation accounts for approximately half to three-fifths of liability, leaving a large role for environment and gene–environment interaction) and several of the highest-confidence genetic risk factors are themselves components of the epigenetic machinery, including chromatin remodelers, histone modifiers, and methylation readers and writers (15). A substantial part of inherited susceptibility is, in effect, a reduced capacity to build and maintain the epigenome, so that, in developmental neurotoxicity, genetic susceptibility and environmental insult are not two separate hazards but two perturbations converging on a single regulatory layer. No disease makes the case for an exposure-indexed, developmentally resolved epigenome more directly.

Framing the sentinel developmentally should not narrow it to a single moment. Brain vulnerability is better understood as a dynamic, life-course neural exposome that extends from development through senescence and is shaped continually by the opposing processes of metaplasticity and neurodegeneration (16). Within that life course, the prenatal window is not a property of the fetus alone but of the maternal–placental–fetal triad, in which endogenous and exogenous stressors interact across trimester-specific exchanges of nutrition and waste to shape the developing neural exposome (17, 18). This places girls’ and women’s health at the center rather than the periphery: matrescence (the life-phase from menarche through menopause across which pregnancies, planned and unplanned, occur) carries transgenerational, life-course consequences for brain health, so a response-epigenome program must be designed for maternal as well as child health (19).

## 3 The space is filling fast: naming the precise gap

It would be a mistake to present this as virgin territory; the honest framing is that adjacent efforts are accelerating and narrowing the unoccupied space, which makes a precise definition of the gap essential. The Pregnancy and Childhood Epigenetics (PACE) consortium coordinates early-life exposure epigenome-wide association studies across many cohorts (20). The Human Early Life Exposome (HELIX)^9^ project (21) linked more than a hundred prenatal and childhood exposures to methylomic and other molecular profiles in over a thousand mother–child pairs, and found that prenatal exposures were captured especially well by child DNA methylation (22). The NIEHS TaRGET II consortium studies exposure-induced epigenomic perturbation and the target-vs-surrogate-tissue problem directly, generating hundreds of multi-omic profiles from mice exposed across life stages and releasing them through a public data portal (23). A 2026 single-cell study mapped how genetic variation and pathogen or chemical exposures distinctively shape the human immune-cell epigenome, separating exposure-associated from genotype-associated differentially methylated regions (24). Population cohorts such as MAMELI are now explicitly designed to track exposome effects through methylation over time (25). And for the developing brain specifically, a human-relevant *experimental* arm already exists: over approximately 18 years the field has moved from no alternative to animal study to a comprehensive developmental-neurotoxicity *in vitro* battery, built on human induced pluripotent stem cell and microphysiological system models, organized around key neurodevelopmental processes and adverse-outcome-pathway networks, and now covered by OECD guidance [(No. 377) 11]. The BrainBow project (26, 27) aims to multiplex these in human brain organoids (28), and validation is on the way under the Validation Qualification Network (VQN)^10^^,^^11^ of the Foundation of the NIH (FNIH)^12^. This is exactly the controlled perturbation capacity that a response-epigenome program needs for causal validation (Section 6), and it is already standardized.11.

These efforts are valuable and should be built upon, not duplicated. But each map either *reference* states, *disease* states, or single exposures in a single tissue, often cross-sectionally, often in a surrogate tissue, often in one species or one cell compartment. The defensible gap is therefore not “*we need a Human Epigenome Project*”; that name is historically occupied by Roadmap and IHEC, and “*HEP*” increasingly denotes the Human Exposome Project. The gap is sharp and unfilled: *a coordinated human atlas of epigenomic response trajectories, indexed by exposure, mixture, dose, developmental window, genotype, cell type, and recovery, and anchored where the public-health need and the mechanistic legibility are greatest, in the developing brain.*

## 4 Defining the object: the human response epigenome

The proposal becomes concrete only if its object is defined precisely. A human response epigenome is the change in cell-type-specific epigenomic state over time following a defined exposure or exposure mixture, conditioned on internal dose, genotype, developmental stage, sex, ancestry, prior exposure history, and time since exposure. The mapping unit is no longer a tissue or cell type alone; it is the product of:

$$\begin{array}{l} \text{cell type}\times \text{tissue}\times \text{exposure}/\text{mixture}\times \text{dose}\times \text{timing}\times \text{duration}\\ \times \text{genotype}\times \mathrm{age}/\mathrm{sex}\times \text{ancestry}\times \text{prior exposure history}\times \text{sampling time}\times \text{recovery state}. \end{array}$$

This single change of unit is what distinguishes the proposal from reference maps. It also forces a useful taxonomy that the field too often blurs. A response-epigenome program must separate (i) *baseline reference* states, (ii) *acute response* states during or immediately after exposure, (iii) *persistent memory* states that endure after exposure ends, (iv) *disease-associated* states, and (v) *recovery or resilience* states after cessation or remediation. These are different objects with different evidentiary status, and conflating them (particularly treating any exposure-associated mark as a stable, inherited memory) is the principal source of overclaiming. The directional hierarchy must likewise be kept explicit: external exposure → internal dose and chemical exposome → molecular sensing and response → epigenomic change → altered cell state → phenotype. Because the broad definition of the exposome folds the body’s responses into the construct, the causal diagram must distinguish exposure, internal dose, response, and consequence, or the proposed mediator ends up defined as part of the exposure it is meant to explain.

For the prenatal window specifically, this mapping unit is instantiated as the maternal–placental–fetal triad (three coupled exposomes (maternal, placental, fetal) sampled across trimester-specific windows), which we propose as the prenatal central unit of the response epigenome (Figure 2) (18, 19).

**Figure 2:**
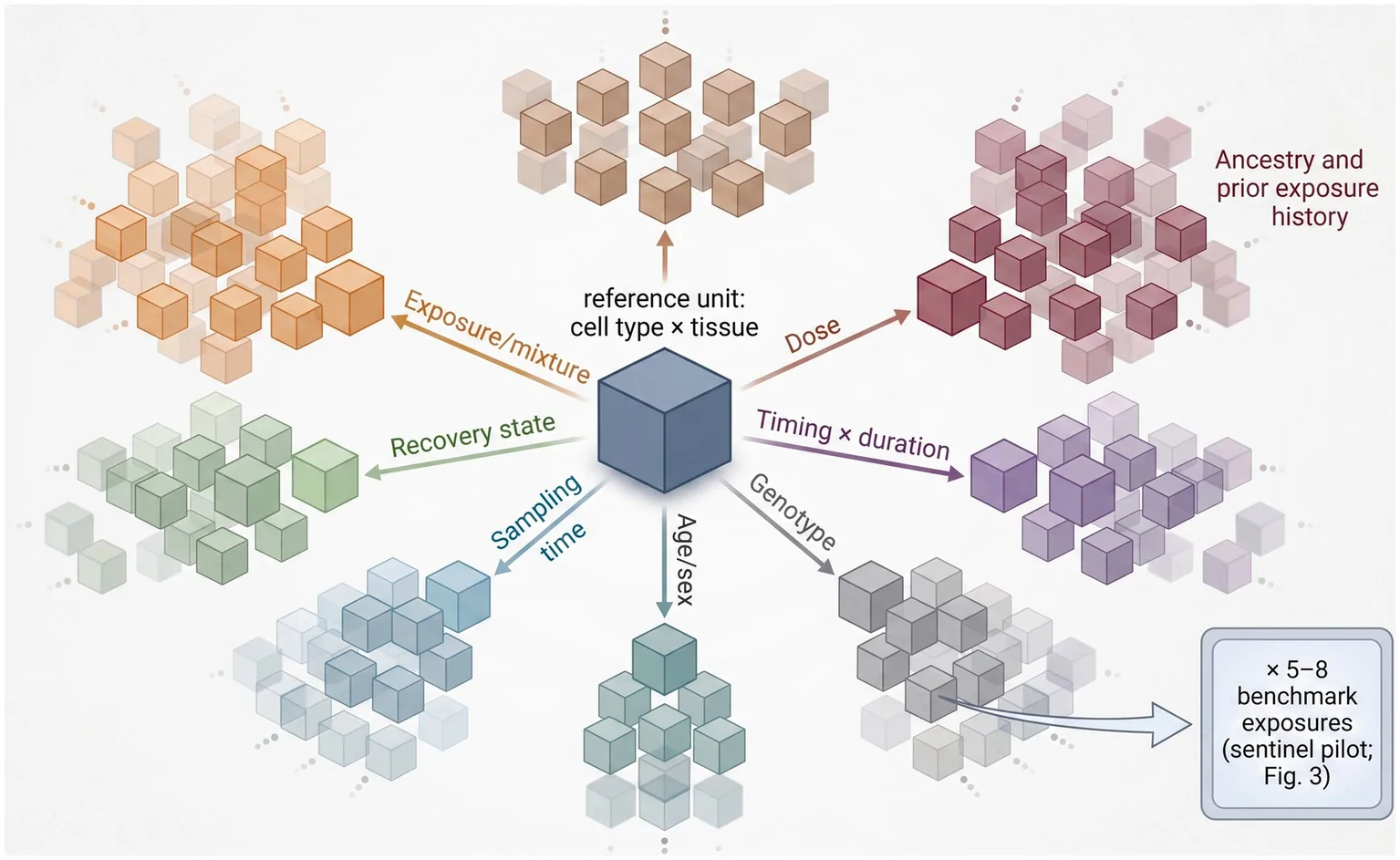
Mapping unit of the response epigenome. Reference atlases are indexed by cell type and tissue (in the inner cube). A response epigenome multiplies this baseline unit by the dimensions that determine how a mark changes after exposure: Exposure/mixture, dose, timing and duration, genotype, age and sex, sampling time, and recovery state, thereby yielding a high-dimensional space that cannot be measured exhaustively. This combinatorial expansion is why the program must proceed with sentinel exposures and a tiered, scalable design (Figure 3 and Table 1) rather than attempting complete coverage.

## 5 A minimum viable response epigenome and a sentinel-exposure pilot

International Human Epigenome Consortium’s lasting methodological contribution was to define what constitutes a *reference* epigenome, which assays, what quality, which replicates, and what metadata (3). The equivalent standard for an *exposure-response* epigenome does not yet exist, and defining it is the most useful thing a perspective can do. Table 1 specifies the components such a standard must cover. The organizing principle is a tiered “*minimum viable response epigenome*”: biospecimen and metadata discipline; population-scale association with rigorous cell-type correction and replication; demonstration of a causal pathway; biological validation in experimental models; and translational deployment, climbing only where a signal earns the investment.

**Table 1:** Components of an exposure-response epigenome standard.

Component	What must be specified
Reference vs. response	separate baseline reference, acute response, persistent memory, disease-associated, and recovery/resilience states
Longitudinal design	Samples before, during, and after exposure, including washout/remediation; cross-sectional studies cannot define trajectories
Exposure synchronization	External measures plus internal-dose biomarkers collected contemporaneously with epigenomic samples
Dose and mixtures	Multiple doses, realistic mixtures, sensitive windows, time courses — not exposed/unexposed labels
Cellular resolution	Population-scale methylation followed by single-cell methylome/accessibility/transcriptome in informative subsets
Genomic constraint	Genome sequencing, meQTLs, allele-specific and response-QTL effects, ancestry- and pangenome-aware analysis
Target–surrogate tissue	Explicit comparison of accessible with disease-relevant tissues, building on TaRGET
Causality	Longitudinal human evidence plus organoid/microphysiological system perturbation, epigenome editing, rescue, replication; two-step Mendelian randomization
Diversity and justice	Global, occupational, and highly exposed communities; structural exposures; benefit-sharing
Infrastructure	Exposure ontologies, assay standards, harmonized metadata, negative controls, versioned pipelines, FAIR and federated analysis
Privacy	Governance for molecular inference of smoking, infection, adversity, occupational and prenatal history
Success criteria	Reproducibility, dose–response, temporal stability, target-tissue concordance, functional consequence, reversibility, public-health utility

Because the exposome is too large to measure everything in everyone, the realistic first step is not exhaustive coverage but a sentinel-exposure pilot (Figure 3). Select five to eight benchmark exposures with strong internal-dose biomarkers and distinct mechanisms, weighted toward developmental neurotoxicity, e.g., organophosphate pesticides, brominated flame retardants, lead, methylmercury, ambient air pollution and combustion products, and an endocrine-active chemical, with an infection or vaccination and a psychosocial-adversity comparator to span the external and social exposome. Sample across prenatal/early-life, adult, and aging windows; include at least one cessation, removal, or remediation setting so that recovery trajectories, not just exposed vs. unexposed contrasts, can be defined. Apply scalable methylation and genotype assays at cohort scale, with single-cell methylome, accessibility, and transcriptome profiling in nested subsets to resolve the cell-composition confounding. Pair every human observation with controlled exposure, washout, and rescue in human organoid or microphysiological systems with functional endpoints for the brain (29, 30), induced-pluripotent stem cell models, and the standardized developmental-neurotoxicity *in vitro* battery organized around key neurodevelopmental processes and adverse outcome pathways (11).

**Figure 3:**
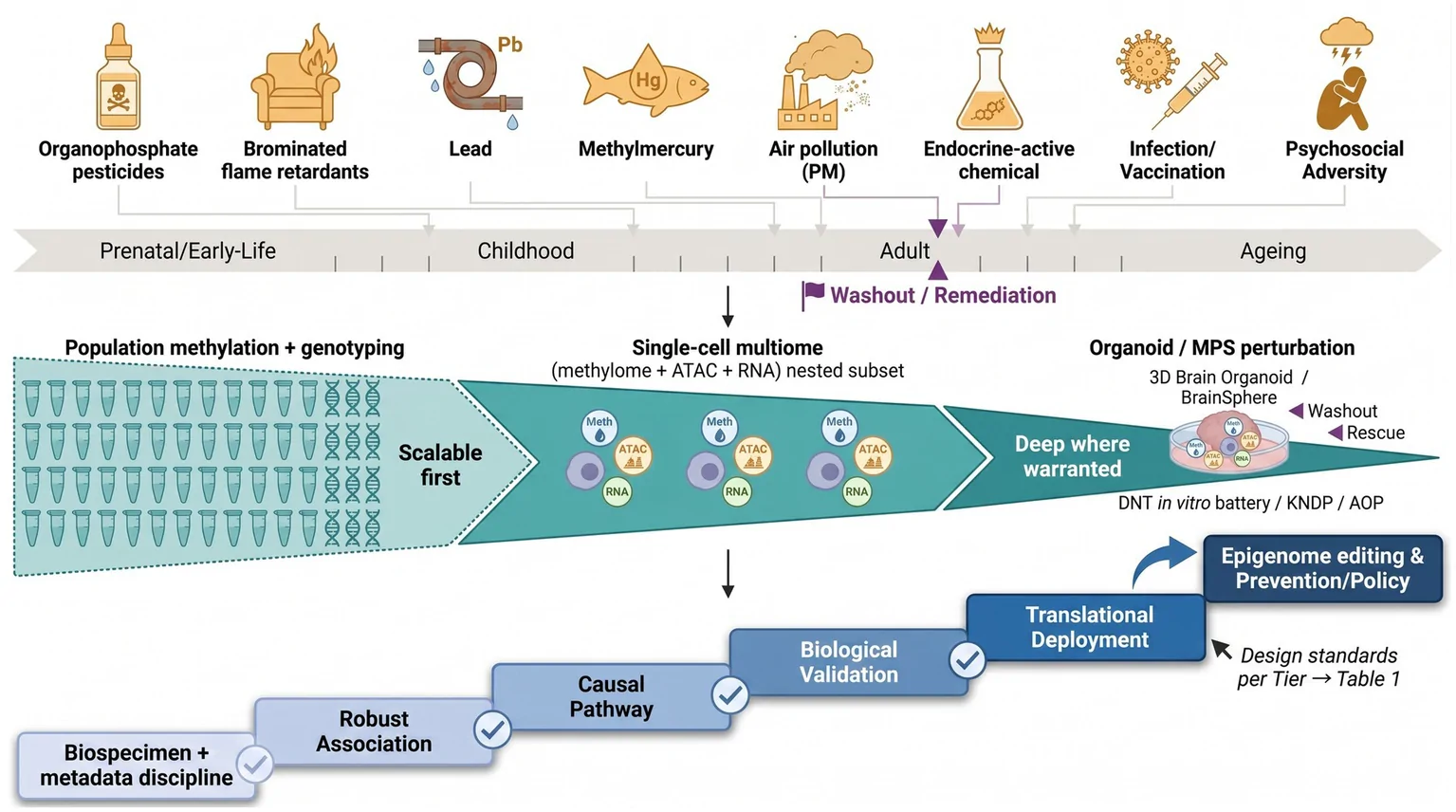
Executable design: Sentinel-exposure pilot and tiered minimum-viable response epigenome. (Top) Five to eight benchmark exposures of distinct mechanisms and with strong internal-dose biomarkers, e.g., organophosphate pesticides, brominated flame retardants, lead, methylmercury, air pollution, and endocrine-active chemicals, plus infection/vaccination and psychosocial-adversity comparators, are studied across prenatal/early-life, adult, and ageing windows, with repeated sampling that includes at least one cessation or remediation setting so that recovery trajectories can be defined. (Middle) Measurement escalates from population-scale methylation and genotyping to single-cell methylome/accessibility/transcriptome in nested subsets to controlled exposure, washout, and rescue in human organoid and microphysiological system models (for the brain, the iPSC-based developmental neurotoxicity *in vitro* battery). (Bottom) Progression is gated by a tiered evidence ladder: Tier 0 biospecimen and metadata discipline; Tier 1 robust association; Tier 2 causal pathway; Tier 3 biological validation; Tier 4 translational deployment. Thereby, only signals meeting predefined criteria advance to epigenome editing and translational use. The design standards each tier must satisfy are specified in Table 1.

Advance only signals that meet predefined evidentiary criteria for targeted epigenome editing (now feasible for locus-specific methylation and chromatin changes (31)) and translational validation. This converts a general design ladder into an executable consortium plan.

## 6 The causal and validation bars

A response atlas is only as useful as the causal claims it can support, and most epigenome-wide associations are cross-sectional and vulnerable to confounding, reverse causation, and cell-composition artifacts. The standard must therefore be explicit and demanding, drawing on two traditions that are rarely combined. From epidemiology comes two-step epigenetic Mendelian randomization, which uses genetic instruments to test whether an exposure causally affects methylation and whether that methylation, in turn, causally affects the outcome (32). meQTLs (methylation Quantitative Trait Loci) are genetic loci, typically SNPs, where inherited DNA sequence variation is statistically associated with differences in DNA methylation levels at specific CpG sites.

DNA methylation levels at specific CpG sites. The meQTLs that constrain the epigenome (33) are precisely the instruments this approach needs, so genomic constraint becomes an analytic asset rather than only a confounder. From toxicology comes the apparatus of evidence-based mechanism evaluation (directed acyclic graphs, adverse outcome pathway networks, and the principle that robust causal claims require both mechanistic and difference-making evidence, neither of which is sufficient alone (34)). The “*meet-in-the-middle*” logic, i.e., intermediate marks associated both retrospectively with exposure and prospectively with disease, sits naturally between the two (35).

Our developmental-neurotoxicity study illustrates both the promise and the discipline required: A human stem-cell experiment exposed brain organoids carrying a heterozygous loss-of-function variant in the chromatin-remodeler gene *CHD8* to the organophosphate chlorpyrifos and its oxon metabolite, and found a synergistic, not merely additive, perturbation: reduced CHD8 protein in the mutant background, disrupted neurite outgrowth, an excitatory/inhibitory neurotransmitter imbalance with lowered dopamine, and shifts in a metabolite panel: the cholinergic system, S-adenosylmethionine and S-adenosylhomocysteine, whose ratio is commonly used as an index of methylation potential, Figure 4), lactate, tryptophan, kynurenic acid, and α-hydroxyglutarate; this was consistent with metabolic alterations reported in ASD patients (15). This is a compelling human-relevant gene– environment interaction, and the concordance with patient data strengthens its translational relevance. It is also, read honestly, a *candidate* epigenetic–metabolic convergence rather than a demonstration of epigenetic mediation: the study measured CHD8 protein, neurodevelopmental endpoints, and selected metabolites, not the methylome, histone marks, or chromatin accessibility downstream of exposure and we ourselves noted that the treatment was designed to model neurotoxicity rather than a realistic exposure scenario and that more detailed analyses are required to clarify mechanism (15). The missing experiments define exactly the standard a response-epigenome program should institutionalize: methylome and chromatin-accessibility profiling across genotype × exposure; temporal ordering of metabolic and chromatin changes; links from altered regions to gene expression; targeted epigenome perturbation or metabolic rescue; and tests of persistence or recovery after washout. A program that holds every candidate to this ladder and that treats epigenetic clocks as composite prognostic predictors rather than direct readouts of the lifetime exposome, mindful that some, such as GrimAge, build an exposure surrogate into the predictor itself (36–38), will produce claims that survive scrutiny.

**Figure 4:**
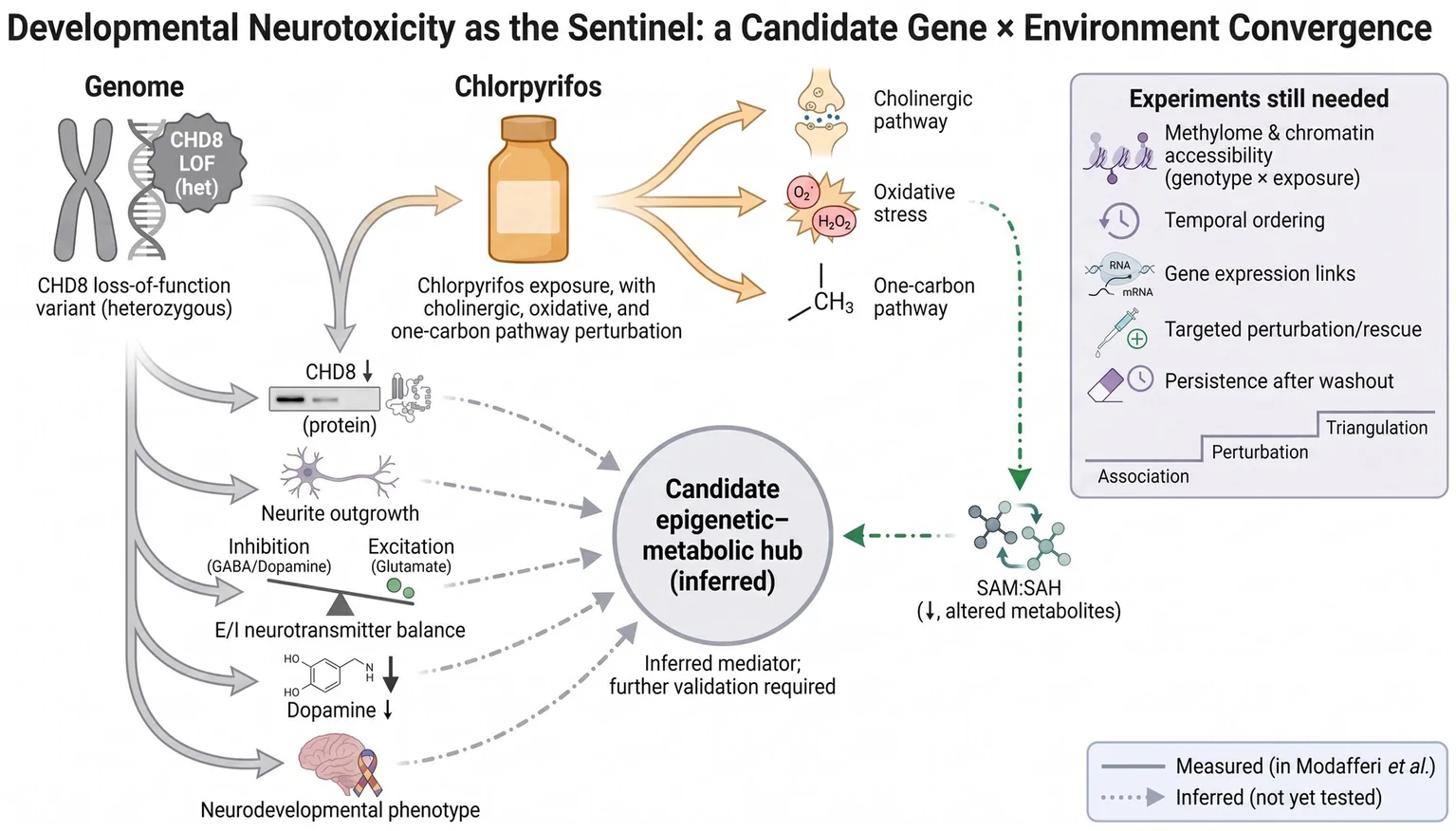
Developmental neurotoxicity as the sentinel case: A candidate gene–environment convergence in a human brain model. In human iPSC-derived brain organoids, a heterozygous loss-of-function variant in the chromatin-remodeler gene *CHD8* (genome side) and exposure to the organophosphate pesticide chlorpyrifos (exposome side) combine *synergistically*, reducing CHD8 protein and perturbing neurite outgrowth, the excitatory/inhibitory neurotransmitter balance (with lowered dopamine), and a metabolite panel that includes the S-adenosylmethionine:S-adenosylhomocysteine (SAM:SAH) pool that sets methylation potential, consistent with metabolic alterations reported in autism. Solid arrows denote relationships measured in the cited study; the central epigenetic–metabolic hub and its link to downstream chromatin changes are inferred and remain to be tested. The boxed experiments (methylome and chromatin-accessibility profiling across *genotype* × *exposure*, temporal ordering, links to gene expression, targeted perturbation or rescue, and persistence after washout) are the standard a response-epigenome program should institutionalize. Based on the experimental system of Modafferi et al. (15).

Two further requirements follow from taking causation seriously. First, the directed acyclic graphs on which this inference depends are only as complete as the disciplines that inform them; connecting knowledge across interrelated fields surfaces additional mediating, moderating, and confounding factors and yields more comprehensive graphs than any single discipline would draw (18). Second, building and interpreting a response epigenome is inherently interdisciplinary, requiring a consortium that spans bench and bedside (from obstetrics, midwifery, nursing, pediatrics, neurology, and pathology to engineering, biophysics, epidemiology, the social sciences, bioethics, and law) (18).

## 7 Ethics, justice, and governance

An exposure-indexed epigenome is, by construction, a record from which a sensitive history can be inferred (e.g., smoking, infection, adversity, occupational and prenatal exposures, and accelerated biological aging). This is not a peripheral concern to be appended at the end of a methods plan; it is a design requirement. Governance, consent, privacy-by-design, and clear limits on molecular inference must be built in from Tier 0. Equity is equally central. A response atlas assembled only from convenience cohorts of European ancestry would entrench existing biases and miss the communities (occupational, highly exposed, and structurally disadvantaged) for whom the public-health stakes are highest; global representation and benefit-sharing are scientific as well as ethical necessities, given the strong dependence of epigenomic state on ancestry and prior exposure.

Equity, in turn, is inseparable from intersectionality. A response-epigenome effort must be a genuine partnership between the Global South and North rather than an extension of research colonialism, so that person-specific and population-health priorities are preserved across diverse communities (39). Its analyses should treat intersecting factors (biological sex, gender identity, race, ethnicity, socio-economic status, and geography) as first-order variables and must span low-, middle-, and high-income settings, recognizing the healthcare deserts that exist within all of them (39, 40). Interdisciplinarity and intersectionality are, in this sense, dual requirements of the same program (40).

The current policy momentum around child health is an opportunity, but it carries a specific risk that this field must name. The biological embedding of exposure can be read, wrongly, as an individual deficit “*your biology shows your exposure history*” and used to shift responsibility from polluters and social structures onto marked individuals. Commentary on the *Make America Healthy Again* initiative has cautioned against exactly this politicization of a health agenda^13^ (12). The corrective is to pair the response epigenome with prevention and structural accountability: its value lies in identifying and removing harmful exposures and in revealing windows for intervention, not in marking the exposed.

## 8 Conclusion: from description to prevention

Reference epigenomes answered where regulatory states existed; exposomics now needs to know how those states change, persist, and recover after exposure. That shift from reference to response is where the next decade of epigenomic public health will be decided, and it is more novel, more falsifiable, and more programmatic than any restatement of the genome–environment bridge. Developmental neurotoxicity is the place to begin: the need is urgent and policy-backed, the developing brain is the most vulnerable and most mechanistically legible target, and human stem-cell models already enable decisive experiments. The epigenome is a principal, durable, cell-state-specific interface between genome and environment, not the only one, and not a turnkey therapy, but it is the layer at which exposure becomes a record we can read and, in time, learn to rewrite. Mapping the human response epigenome, beginning with the developing brain, would turn that recognition into prevention.

Realizing this in practice is a transdisciplinary task in which patients, families, clinicians, scientists, and communities share decisions about how the resulting tools are used (41). What that task needs first is not consensus but a standard: an agreed mapping unit, a small set of benchmark exposures, and a tiered evidence ladder that a response epigenome must climb before it informs policy. Table 1. and Figure 3 are a first draft of that standard. The developing brain is where getting it right matters most, and where the work can begin now.

## Footnotes

1
https://ihec-epigenomes.org

2
https://www.iarc.who.int

3
https://www.humancellatlas.org

4
https://globalexposomeforum.org

5
https://www.humancellatlas.org/event/towards-a-human-exposome-cell-atlasdecember-2025/

6
https://www.humancellatlas.org/event/towards-a-human-exposome-atlas-march-2026/

7
https://www.cdc.gov/autism/data-research/index.html

8
https://www.niehs.nih.gov/news/factor/2025/2/feature/4-feature-epigenetics-and-theexposome

9
https://cordis.europa.eu/project/id/308333/reporting

10
https://commonfund.nih.gov/complementarie/vqn

11
https://fnih.org/our-programs/validation-qualification-network-design-phase/namspilot-projects/

12
https://fnih.org/our-programs/validation-qualification-network-design-phase/

13
https://www.whitehouse.gov/presidential-actions/2025/02/establishing-thepresidents-make-america-healthy-again-commission/
